# RdDM-Dependent Epigenetic Regulation Coordinates Systemic Immunity and Compatibility with *Trichoderma atroviride* in *Arabidopsis thaliana*

**DOI:** 10.3390/microorganisms14040914

**Published:** 2026-04-18

**Authors:** Maria Montserrat Rosendo-Vargas, Valeria Ávila-Castillo, Kumari Rashmi, Sergio Casas-Flores

**Affiliations:** Instituto Potosino de Investigación Científica y Tecnológica, A. C. (IPICYT), División de Biología Molecular, Camino a la Presa San José No. 2055, Colonia Lomas 4ª Sección, San Luis Potosí C.P. 78216, Mexico; maria.rosendo@ipicyt.edu.mx (M.M.R.-V.); valeria.avila@ipicyt.edu.mx (V.Á.-C.); kumari.rashmi@ipicyt.edu.mx (K.R.)

**Keywords:** *Arabidopsis*, *Trichoderma*, RNA-directed DNA methylation (RdDM), DICER-LIKE (DCL) proteins, ARGONAUTE 9 (AGO9), DNA methyltransferases, systemic resistance, plant–microbe interactions

## Abstract

Epigenetic regulation plays a central role in modulating plant immune responses and interactions with beneficial microbes. In this study, we investigated the contribution of RNA-directed DNA methylation (RdDM) components—DCL3; AGO9; DCL1; and the de novo DNA methyltransferases CMT3, DRM1, and DRM2—to the interaction between *Arabidopsis thaliana*, *Trichoderma atroviride*, and foliar pathogens. We show that DCL3 and AGO9 differentially regulate basal and inducible immunity, negatively affecting resistance to the necrotrophic fungus *Botrytis cinerea*, while promoting defense against the hemibiotrophic bacterium *Pseudomonas syringae* pv. *tomato* DC3000. Transcriptional analyses revealed that RdDM components modulate the balance between jasmonic acid/ethylene (JA/ET) and salicylic acid (SA) signaling pathways, influencing the amplitude and coordination of defense responses. In addition, DCL3 and DCL1 appear to be required for the full expression of *T. atroviride*-mediated systemic resistance, whereas AGO9 and DNA methyltransferases contribute to efficient root colonization. Notably, mutants in these pathways displayed enhanced basal resistance but impaired responsiveness to beneficial microbial signals, revealing a trade-off between constitutive defense activation and inducible systemic protection. Consistent with this, alterations in RdDM components were also associated with changes in plant growth dynamics under specific conditions, supporting a role for epigenetic regulation in coordinating growth–defense trade-offs. Together, our findings support a model in which epigenetic regulation controls defense responsiveness, enabling plants to balance immune activation, growth and compatibility toward beneficial microbes.

## 1. Introduction

Plants have evolved a wide array of defense mechanisms to counteract pathogen invasion. The first two layers involve physical and chemical barriers that function as the plant’s primary line of defense. Physical barriers include structural components such as trichomes, wax cuticles, and rigid cell walls, which restrict pathogen entry and colonization. In parallel, chemical barriers comprise a diverse arsenal of secondary metabolites with antimicrobial properties that inhibit pathogen establishment and contribute to basal immunity [1,2,3].

When pathogenic microbes succeed in breaching these barriers, plant defense relies on sophisticated cellular recognition mechanisms [4]. This response is initiated by the detection of pathogen-associated molecular patterns (PAMPs) through membrane-localized pattern recognition receptors (PRRs), leading to the activation of PAMP-triggered immunity (PTI). In response, pathogens have evolved effector proteins that suppress PTI upon delivery into host cells, a process known as effector-triggered susceptibility (ETS). As a countermeasure, plants deploy effector-triggered immunity (ETI), a more robust defense response mediated by intracellular resistance (R) proteins that specifically recognize pathogen effectors. This recognition often results in a localized hypersensitive response (HR) and the establishment of a primed state, enabling the plant to respond more rapidly and effectively to future biotic or abiotic stress [5]. In parallel, PAMP perception can also activate long-distance defense signaling pathways, including systemic acquired resistance (SAR), which is primarily mediated by salicylic acid (SA), and induced systemic resistance (ISR), which relies on jasmonic acid (JA) and ethylene (ET) signaling [4,6]. Beyond these classical hormonal pathways, emerging evidence points to a critical role for small RNAs (sRNAs) and chromatin remodeling in fine-tuning the transcriptional landscapes associated with both PTI and ETI, thereby adding an epigenetic layer of control to plant immune responses [7].

Epigenetic regulation of plant immunity involves gene silencing mechanisms mediated by sRNAs of 21–24 nucleotides (nt), which guide target mRNA degradation, translational repression, or DNA methylation via the RNA-directed DNA methylation (RdDM) pathway [4,8]. In this pathway, RNA Polymerase IV (Pol IV) generates single-stranded RNA (ssRNA) transcripts, which are subsequently converted into double-stranded RNAs (dsRNA) by RNA-dependent RNA Polymerase 2 (RDR2). These dsRNAs are then processed by Dicer-like 3 (DCL3) into 24-nt sRNAs. The resulting sRNAs are incorporated into ARGONAUTE proteins, primarily AGO4, AGO6, or AGO9, forming effector complexes that mediate sequence-specific targeting. In association with the de novo DNA cytosine-5-methyltransferase (DRM2), these complexes are recruited to scaffold transcripts generated by RNA Polymerase V (Pol V), thereby directing cytosine methylation at homologous genomic *loci* [8,9,10,11,12]. Recent studies indicate that this epigenetic machinery, beyond its canonical role in transposon silencing and genome integrity, also contributes to the regulation of plant immune responses. Specifically, components of the RdDM pathway have been implicated in the transcriptional reprogramming of defense-related genes upon microbial perception, suggesting a critical interface between epigenetic control and environmental responsiveness [13].

Beneficial microbes, including plant growth-promoting fungi (PGPF) such as *Laccaria bicolor* and *Trichoderma* spp., can enhance plant immunity by modulating defense readiness through interconnected hormonal and epigenetic signaling pathways [14,15]. *Trichoderma* spp. comprises a group of filamentous fungi widely recognized for their mycoparasitic capabilities, enabling them to antagonize a broad range of soilborne pathogens. Beyond their direct biocontrol activity, these fungi contribute to plant health by promoting root development and improving nutrient acquisition [16,17].

Upon root colonization, *Trichoderma* simultaneously activates defense-related genes associated with the SA and JA/ET signaling pathways, thereby enhancing protection against both biotrophic and necrotrophic pathogens [18,19]. This response is largely mediated by an array of microbe-associated molecular patterns (MAMPs) and proteinaceous elicitors, including polygalacturonases, xylanases, ceratoplatanins, cellulases, swolenins, and hydrolases, which stimulate systemic resistance in the host [20]. Additionally, *Trichoderma* is known to establish a primed physiological state, enabling a faster and more robust defense upon subsequent pathogen challenge [21].

The role of key components of the RdDM pathway in plant immunity has been increasingly supported by genetic evidence in *Arabidopsis thaliana*. For instance, AGO4 has been shown to modulate specific aspects of the disease response against the hemibiotrophic bacterium *Pseudomonas syringae* (*Pst* DC3000) [22]. Similarly, Pol V has emerged as a critical factor in mediating resistance against necrotrophic fungal pathogens such as *Botrytis cinerea* and *Plectosphaerella cucumerina* [23]. Both AGO4 and RDR2 contribute to Arabidopsis immunity against *B. cinerea*, indicating that canonical RdDM components play a broader role in defense across diverse pathogen lifestyles [24]. Moreover, AGO4, AGO6, POL V, and RDR2 have been shown to be required for efficient root colonization by *Trichoderma atroviride*, highlighting a potential link between RdDM-mediated chromatin regulation and the establishment of beneficial plant–microbe interactions [24]. However, despite increasing evidence linking RdDM components to both plant immunity and beneficial root colonization, it remains unclear how this epigenetic machinery coordinates the balance between defense activation and mutualistic compatibility, particularly in response to pathogens with contrasting lifestyles. This gap is especially relevant given the potential trade-off between enhanced resistance and the establishment of beneficial interactions.

In this study, we investigated the functional role of the RNA-directed DNA methylation (RdDM) pathway in the tripartite interaction among *A. thaliana*, foliar pathogens with contrasting lifestyles, and the beneficial fungus *T. atroviride*. Here, we addressed this knowledge gap by dissecting how key RdDM components, including DCL3, AGO9, DCL1, and de novo DNA methyltransferases, functionally integrate systemic immunity and beneficial fungal accommodation. To address this, we combined phenotypic, molecular, and colonization analyses using Arabidopsis mutant lines impaired in these components. We first evaluated plant growth promotion in response to *T. atroviride*, followed by pathogen challenge assays with *B. cinerea* and *P. syringae* DC3000 after root treatment. In parallel, we quantified the expression of key marker genes associated with the SA- and JA/ET-dependent pathways to assess transcriptional reprogramming in RdDM-deficient backgrounds. Finally, we performed root colonization assays to determine the extent of *T. atroviride* establishment in these genotypes, revealing how epigenetic regulators balance systemic immunity with mutualistic compatibility.

## 2. Materials and Methods

### 2.1. Organisms and Growth Conditions

*Arabidopsis thaliana* ecotype Columbia (Col-0) and the following insertional mutant lines were used in this study: *dcl1-9* [25], *dcl3-1* [26], *cmt3-11* [27], *drm1-2/drm2-2* [28] and *cmt3-11/drm1-2/drm2-2* (*cdd*) [27], all obtained from Arabidopsis Biological Resource Center (ABRC). The Arabidopsis *ago9-2* mutant line [29] was kindly provided by Dr. Jean Philippe Vielle-Calzada. Seeds were surface sterilized using 75% ethanol for 4 min, followed by a treatment with 20% commercial bleach (sodium hypochlorite) for 8 min, and rinsed three times with sterile distilled water. The seeds were stratified at 4 °C for 2 days and then germinated in a controlled growth chamber under a 16 h light/8 h dark photoperiod at 22 °C. One-day-old seedlings were transferred to Petri dishes containing 0.5× Murashige and Skoog (MS) medium and grown for an additional 10 days under the same conditions. *Trichoderma atroviride* strain IMI206040 was cultured on Potato Dextrose Agar (PDA) at 25 °C for 3–7 days, depending on the experimental requirements. *Botrytis cinerea* strain B05.10 [30] was grown on PDA for 14 days at 25 °C. *Pseudomonas syringae* pv. *tomato* DC3000 [31] was propagated in King’s B medium supplemented with rifampicin (50 μg/mL) and incubated at 28 °C for 24 h.

### 2.2. Expression Analysis of Arabidopsis Genes

The ten-day-old Arabidopsis seedlings were root-inoculated with *T. atroviride* conidia, and samples were collected at 72 h post-treatment (hpt). Roots and leaf tissues were harvested and immediately frozen in liquid nitrogen for subsequent homogenization. Total RNA was extracted using a modified cetyltrimethylammonium bromide (CTAB) protocol [32]. Briefly, homogenized tissue was suspended in 200 μL of extraction buffer (200 mM Tris-HCl, pH 8.0; 250 mM NaCl; 25 mM EDTA; 0.5% SDS) and 400 μL of 2× CTAB (100 mM Tris-Cl, pH 8.0; 1.4 M NaCl; 20 mM EDTA; 2% CTAB), followed by incubation on ice for 20 min. RNA was subsequently purified by phenol–chloroform extraction. RNA concentration and quality were determined using an Epoch Microplate Spectrophotometer (BioTek Instruments, Winooski, VT, USA). Complementary DNA (cDNA) synthesis was performed using SuperScript II reverse transcriptase (Invitrogen, Carlsbad, CA, USA), following the manufacturer’s instructions. Gene expression analysis was performed for the defense-related genes *PR-1a*, *ICS1*, *PR5*, *PDF1.2*, *PR4*, and *LOX2*, using gene-specific primer pairs, with *ACTIN2 (ACT2)* as the reference gene. Primer sequences (forward and reverse) used for each gene are provided in Supplementary Table S1. Quantitative reverse transcription PCR (RT-qPCR) was carried out using 200 ng of cDNA and SYBR Green Lo-ROX qPCR kit, according to the manufacturer’s instructions. Gene expression levels were normalized to the *ACTIN2* (*ACT2*) housekeeping gene, and the relative expression was calculated using the 2^−ΔΔCt^ method [33].

### 2.3. Arabidopsis Growth Promotion Mediated by T. atroviride

The seeds were stratified at 4 °C for 2 days and subsequently sown in pots containing a peat moss-based substrate. The plants were maintained in a controlled growth chamber under a 16 h light/8 h dark photoperiod at 21 °C. *T. atroviride* was cultured on PDA for 7 days at 25 °C. Conidia were harvested by flooding the plates with 1 mL of sterile distilled water and quantified using a Neubauer hemocytometer. A conidial suspension was prepared at a concentration of 1 × 10^6^ conidia/mL in 0.3× MS medium. Eight-day-old Arabidopsis seedlings were treated by soil drench with the *T. atroviride* conidia suspension, while control plants received an equal volume of conidia-free 0.3× MS medium. At 18 days post-treatment (dpt), rosette development was documented using the Lab ScanalyzerTS imaging platform (LemnaTec GmbH, Aachen, Germany), and rosette area was quantified. Roots from the treated and control plants were carefully washed and dried at 65 °C for 24 h. Whole plants were subsequently weighed to determine total dry biomass.

### 2.4. Arabidopsis Resistance Assay Against Foliar Pathogens

To assess disease resistance, *B. cinerea* and *P. syringae* pv. tomato DC3000 (*Pst* DC3000) were used as the representative necrotrophic and hemibiotrophic pathogens, respectively. *B. cinerea* was cultured under the conditions previously described, and a conidial suspension was prepared at a concentration of 5 × 10^5^ conidia/mL in phosphate buffer (137 mM NaCl, 2.7 mM KCl, 10 mM Na_2_HPO_4_, and 1.8 mM KH_2_PO_4_; pH 7.4, adjusted with HCl). At 15 dpt with *T. atroviride*, three fully expanded leaves per plant were drop-inoculated with 20 μL of the fungal suspension. Four days post-infection (dpi), lesion development was imaged using the Lab ScanalyzerTS platform (LemnaTec GmbH), and lesion area was quantified using the ImageJ software (v.1.52a).

For *Pst* DC3000, bacterial cultures were grown as previously described. A suspension containing 5 × 10^6^ CFU/mL in sterile distilled water supplemented with 0.0083% (*v*/*v*) Silwet L-77 was prepared. At 72 h post-treatment with *T. atroviride*, roots from both mock- and *Trichoderma*-treated plants were excised at the site corresponding to the fungal contact region to prevent continued interaction. The mock-treated plants were subjected to the same excision procedure to ensure consistency. Subsequently, the aerial parts of the plants were immersed in 20 mL of bacterial suspension for 3 min. The plants were then transferred to Petri dishes containing 0.5× MS medium and maintained under standard growth conditions. Foliar symptoms were documented four dpi using the Lab ScanalyzerTS system. To determine bacterial proliferation, leaves were collected and weighed, and surface-sterilized by immersion in 5 mL of 5% H_2_O_2_ for 3 min, followed by three rinses with sterile distilled water. Tissues were homogenized, and serial dilutions were plated onto King’s B agar supplemented with rifampicin (50 μg/mL). The plates were incubated at 28 °C for 48 h, after which colony-forming units per gram of tissue (CFU/g) were quantified.

### 2.5. Root Colonization Assay

To assess root colonization by *T. atroviride*, ten-day-old Arabidopsis seedlings were submerged for 5 min in 20 mL of phosphate-buffered saline (PBS) containing 1 × 10^7^ conidia/mL of *T. atroviride*. Following inoculation, the seedlings were transferred to Petri dishes containing sterile filter paper moistened with 3 mL of sterile distilled water and incubated at 25 °C for 48 h. After incubation, roots were carefully harvested, weighed, and surface-sterilized by immersion in 1% (*v*/*v*) commercial bleach (sodium hypochlorite) for 2 min, followed by three rinses with sterile distilled water. The surface-sterilized roots were homogenized in a porcelain mortar, and serial dilutions of the homogenate were plated onto PDA supplemented with 0.5% (*v*/*v*) Triton X-100 (Sigma-Aldrich, St. Louis, MO, USA). This detergent was included to limit hyphal spread and improve colony separation, thereby facilitating accurate CFU quantification rather than inhibiting fungal growth. The plates were incubated at 25 °C for 48 h. After incubation, CFUs were counted and expressed as CFU per 100 mg of fresh root tissue. *Trichoderma* colonies were identified based on characteristic macroscopic morphology, including mycelial texture and conidial pigmentation.

### 2.6. Statistical Analysis

All the statistical analyses were performed using GraphPad Prism version 9.0.0 (GraphPad Software, San Diego, CA, USA, www.graphpad.com). One-way analysis of variance (ANOVA) followed by Tukey’s multiple comparisons test was used to compare means in Arabidopsis growth promotion and resistance assays. For gene expression analyses, two-way ANOVA followed by Tukey’s multiple comparisons test was applied to assess the effects of treatment (mock vs. *Trichoderma*) and genotype (Col-0 vs. mutants), including their interaction. Differences in root colonization between Col-0 and mutant lines were evaluated using an unpaired two-tailed Student’s *t*-test. For each experiment, three independent biological replicates were analyzed, each consisting of pooled tissue from multiple plants. All the experiments were independently repeated twice, with 30–40 plants per treatment (n = 30–40). Data are presented as mean ± standard error (SE). Differences were considered statistically significant at *p* < 0.05. In figures, different letters indicate statistically significant differences, whereas groups sharing the same letter are not significantly different.

## 3. Results

### 3.1. RNA Silencing and De Novo DNA Methylation Components Are Differentially Required for Arabidopsis Growth and Its Promotion by T. atroviride

**Figure 1:**
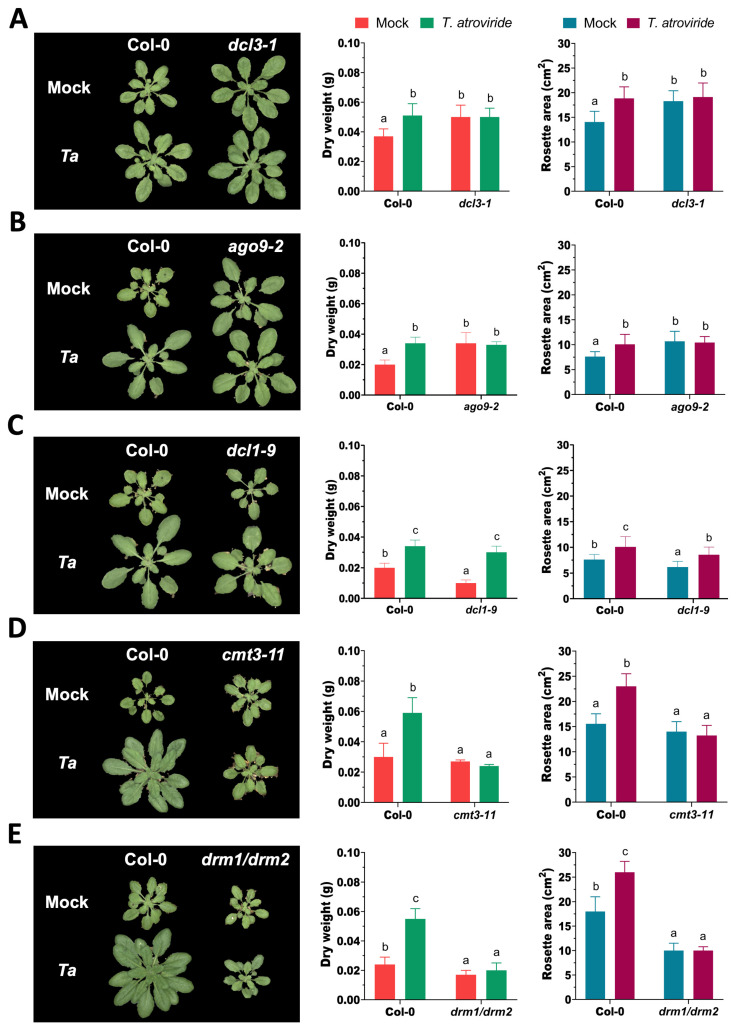
DCL3 and AGO9 act as negative modulators of plant growth in *Arabidopsis thaliana* under mock conditions. Eight-day-old seedlings of Col-0, *dcl3-1* (**A**), *ago9-2* (**B**), *dcl1-9* (**C**), *cmt3-11* (**D**), and *drm1-2/drm2-2* (**E**) were root-treated with *T. atroviride* conidia (*Ta*) or mock-treated with 0.3× MS medium. Representative images were taken at 18 days post-treatment (dpt). Plant growth was assessed by dry weight and rosette area, and quantified using an analytical balance and the ScanalyzerTS imaging platform, respectively. Experiments were independently repeated twice with similar results, using 30 plants per treatment. Error bars represent standard deviation (SD). Different letters indicate statistically significant differences as determined by one-way ANOVA followed by Tukey’s multiple comparison test (*p* < 0.05).

To elucidate the role of the RNA silencing and DNA methylation pathways in *T. atroviride*-induced growth promotion, we evaluated the growth phenotype of *A. thaliana* wild-type (Col-0) and mutant lines impaired in *DCL3* (*dcl3-1*), *AGO9* (*ago9-2*), *DCL1* (*dcl1-9*), *CMT3* (*cmt3-11*), and *DRM1*/*DRM2* (*drm1-2/drm2-2*). Under mock conditions, the *dcl3-1* and *ago9-2* mutants exhibited significantly enhanced biomass accumulation relative to Col-0, suggesting that DCL3 and AGO9 may act as negative modulators of growth under these conditions (Figure 1A,B). However, these mutants did not show further biomass increase upon the *T. atroviride* treatment, and their growth under fungal inoculation was comparable to that of Col-0 (Figure 1A,B). This lack of responsiveness indicates that DCL3 and AGO9 may contribute to the perception or integration of *T. atroviride*-mediated growth-promoting signals in a context-dependent manner. In contrast, *dcl1-9* mutants exhibited reduced growth under mock conditions but displayed a significant biomass increase upon the *T. atroviride* treatment, comparable to that observed in the treated Col-0 plants (Figure 1C). These findings suggest that DCL1 acts as a positive regulator of vegetative growth and contributes to the responsiveness of Arabidopsis to beneficial microbial signals, though its contribution appears to be conditional rather than strictly required for growth promotion by *T. atroviride*. Finally, consistent with previous findings [24], *cmt3-11* single and *drm1/drm2* double mutants exhibited growth phenotypes comparable to those of *cmt3-11/drm1-2/drm2-2* (*cdd*) triple mutant, with no significant differences between the mock and treated conditions (Figure 1D,E). All three genotypes displayed reduced rosette size under control conditions and failed to respond to *T. atroviride*-induced growth promotion. These results indicate that the de novo DNA methyltransferases CMT3, DRM1, and DRM2 are required for both basal plant growth and the growth-promoting response to *Trichoderma*. The consistent lack of responsiveness across these mutants supports a central role for the de novo methylation pathway in enabling the plant’s capacity to perceive or transduce growth-promoting signals triggered by beneficial fungi under the tested conditions.

### 3.2. DCL3, AGO9, and De Novo DNA Methyltransferases Repress Basal Immunity to Botrytis cinerea, While DCL1 Is Required for the Full Activation of Trichoderma-Induced Systemic Resistance

**Figure 2:**
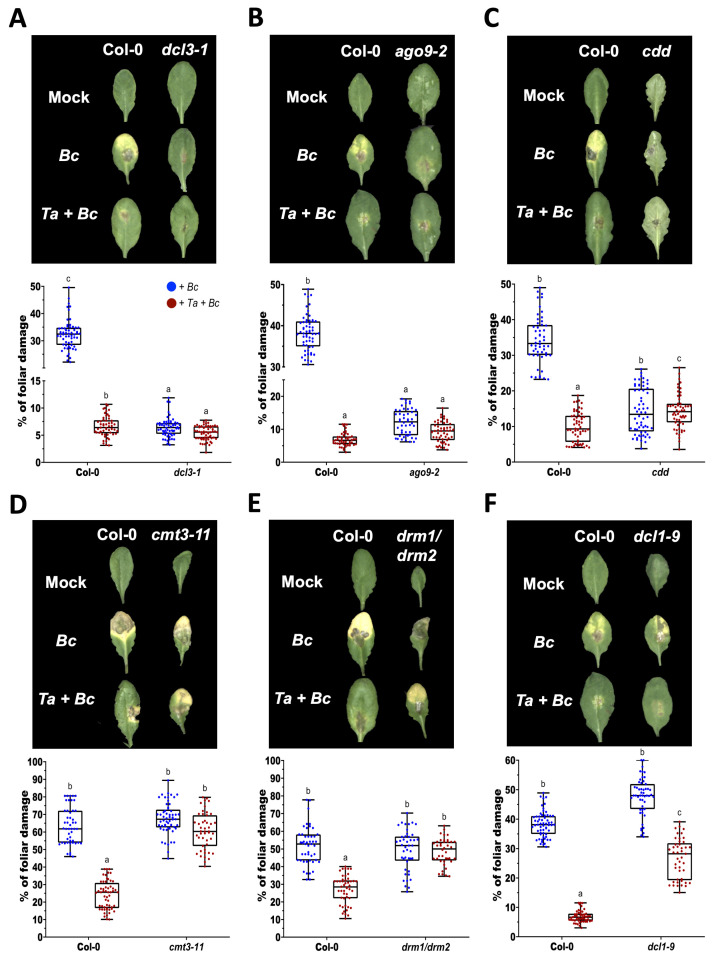
Contribution of RNA silencing and DNA methylation components to *Botrytis cinerea* resistance and *Trichoderma atroviride*-induced systemic resistance in *Arabidopsis thaliana.* Eight-day-old Arabidopsis seedlings were root-inoculated with *T. atroviride* conidia (*Ta*) or mock-treated with buffer. At 15 days post-treatment, the plants were challenged with *B. cinerea* (*Bc*) by drop-inoculating three leaves per plant. Representative images were taken at 4 dpi for Col-0, *dcl3-1* (**A**), *ago9-2* (**B**), *cdd* (**C**), *cmt3-11* (**D**), *drm1/drm2* (**E**) and *dcl1-9* (**F**). Disease severity was quantified as the percentage of foliar area showing necrosis. The *cdd* mutant corresponds to the combined loss of *CMT3*, *DRM1*, and *DRM2*. Experiments were independently repeated twice with similar results, using 20 plants per treatment. Error bars represent standard deviation (SD). Different letters indicate statistically significant differences as determined by one-way ANOVA followed by Tukey’s multiple comparison test (*p* < 0.05).

To evaluate the involvement of DCL3, AGO9, DCL1, and de novo DNA methyltransferases in *T. atroviride*-induced systemic resistance against the necrotrophic pathogen *Botrytis cinerea*, we conducted infection assays in Col-0 and the corresponding mutant lines with or without *T. atroviride* pre-treatment. As expected, *T. atroviride* pre-treatment significantly reduced foliar damage in the Col-0 plants (Figure 2A). In contrast, *dcl3-1*, *ago9-2*, and the *cmt3-11/drm1-2/drm2-2* (*cdd*) triple mutant exhibited enhanced basal resistance to *Botrytis* under mock conditions relative to Col-0. However, the *T. atroviride* pre-treatment did not further increase resistance in these lines, indicating that DCL3, AGO9, and the combined activity of de novo methyltransferases act as negative regulators of basal immunity. Because these mutants already display elevated basal resistance, it is not possible to determine whether these components are also required for *T. atroviride*-induced systemic resistance. Conversely, the *cmt3-11* and *drm1/drm2* mutants exhibited susceptibility to *Botrytis* comparable to Col-0 under mock conditions but failed to respond to *T. atroviride* pre-treatment (Figure 2D,E), indicating that CMT3 and DRM1/DRM2 are dispensable for basal resistance but required for the induction of *T. atroviride*-mediated systemic resistance. Notably, the *dcl1-9* mutants displayed susceptibility similar to Col-0 under mock conditions. Although the *T. atroviride* treatment significantly reduced disease symptoms, the level of protection was lower than in Col-0 (Figure 2F). These findings indicate that DCL1 contributes to basal immunity and is required for the full activation of *T. atroviride*-induced systemic resistance.

### 3.3. DCL3, AGO9, CDD, and DCL1 Differentially Regulate Transcriptional Responses of the JA/ET Pathway in Arabidopsis upon T. atroviride Treatment

**Figure 3:**
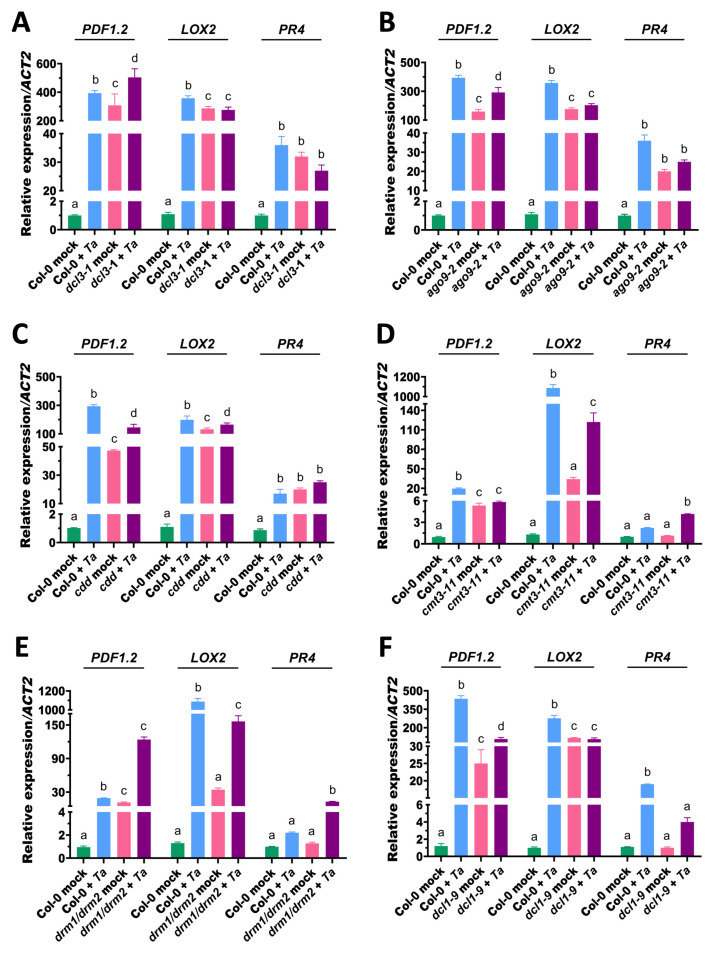
Expression profiles of JA/ET (*PDF1.2*), JA (*LOX2*), and ET (*PR4*) marker genes in Arabidopsis mutants defective in RNA silencing and DNA methylation pathways. Ten-day-old Arabidopsis seedlings were root-inoculated with *T. atroviride* conidia (*Ta*) or mock-treated, and *PDF1.2*, *LOX2* and *PR4* expression was quantified at 72 h post-treatment by RT-qPCR. Gene expression was analyzed in the *dcl3-1* (**A**), *ago9-2* (**B**), *cdd* (**C**), *cmt3-11* (**D**), *drm1/drm2* (**E**) and *dcl1-9* (**F**) mutants. Expression levels were normalized to *ACT2* and calculated relative to the corresponding mock-treated control. Each genotype was analyzed in an independent experiment with its own Col-0 control, which is shown for comparison across panels and may, therefore, appear duplicated. The *cdd* line corresponds to the triple mutant for *CMT3*, *DRM1*, and *DRM2*. Data represent the mean standard deviation (SD) of two independent biological experiments, each including 40 plants per treatment. Different letters indicate statistically significant differences among the treatments as determined by two-way ANOVA followed by Tukey’s multiple comparison test (*p* < 0.05).

To explore whether the altered disease phenotypes in RdDM and silencing mutants were associated with transcriptional reprogramming of the jasmonic acid/ethylene (JA/ET) pathway, we analyzed the expression of *PDF1.2* (JA/ET marker), *LOX2* (JA marker), and *PR4* (ET marker) in Col-0 and mutant lines at 72 h post-treatment (hpt) with *T. atroviride*. In the *dcl3-1* mutant, *PDF1.2*, *LOX2*, and *PR4* were upregulated under mock conditions; however, following the *T. atroviride* treatment, their expression levels were generally lower than in Col-0, with the exception of *PDF1.2* (Figure 3A), indicating an attenuated transcriptional response. Similarly, *ago9-2* displayed elevated expression of the three genes under mock conditions but showed reduced induction after the *T. atroviride* treatment compared to Col-0 (Figure 3B), consistent with a defense plateau likely resulting from pre-activated signaling.

In the *cdd* mutants, JA/ET marker genes were strongly upregulated under mock conditions relative to Col-0. However, upon the *T. atroviride* treatment, Col-0 exhibited robust induction of all three genes, whereas *cdd* showed significantly reduced expression of *PDF1.2* and *LOX2*. *PR4* expression remained largely unchanged in *cdd* across both conditions, reaching levels comparable to Col-0 after the treatment. This pattern is consistent with the *Botrytis*-resistant phenotype of the mutant and suggests constitutive activation of JA/ET signaling coupled with impaired inducible transcriptional reprogramming. In the *cmt3-11* and *drm1/drm2* mutants, *PDF1.2* and *LOX2* were upregulated under mock conditions relative to Col-0, indicating partial pre-activation of JA/ET signaling (Figure 3D,E). In both mutants, *PR4* remained at basal levels under mock conditions but was strongly induced after the *T. atroviride* treatment, exceeding Col-0 levels. These results indicate that transcriptional responsiveness to *T. atroviride* is altered rather than abolished in these backgrounds, which may explain their impaired systemic resistance to *B. cinerea*.

Finally, in *dcl1-9*, *PDF1.2* and *LOX2* were elevated under mock conditions compared to Col-0. After the *T. atroviride* treatment, Col-0 exhibited strong induction of both genes, whereas in *dcl1-9*, *PDF1.2* showed only a modest increase and *LOX2* remained unresponsive (Figure 3F). *PR4* levels also remained low and uninduced in *dcl1-9*, in contrast to Col-0, which showed significant upregulation after *T. atroviride* exposure (Figure 3F). These findings indicate that DCL1 is required not only for basal activation but also for full JA/ET gene induction in response to beneficial microbes. Collectively, these results demonstrate that the DCL3, AGO9, and CDD components primarily modulate basal JA/ET gene expression, whereas DCL1 is essential for effective transcriptional priming and full *T. atroviride*-mediated activation of these defense pathways.

### 3.4. DCL3 and AGO9 Promote Defense Against Pst DC3000, While DCL1 and De Novo DNA Methyltransferases Constrain SA Signaling and T. atroviride-Induced Protection

To elucidate the role of RNA silencing and DNA methylation components in systemic resistance against the hemibiotrophic bacterium *P. syringae* pv. *tomato* DC3000 (*Pst* DC3000), we assessed disease progression in *A. thaliana* wild-type (Col-0) and mutant lines (*dcl3-1*, *ago9-2*, *dcl1-9, cmt3-11, drm1/drm2* and *cdd*), with or without *T. atroviride* pre-treatment. Four days post-infection, foliar symptoms and bacterial load (colony-forming units, CFU) were quantified.

**Figure 4:**
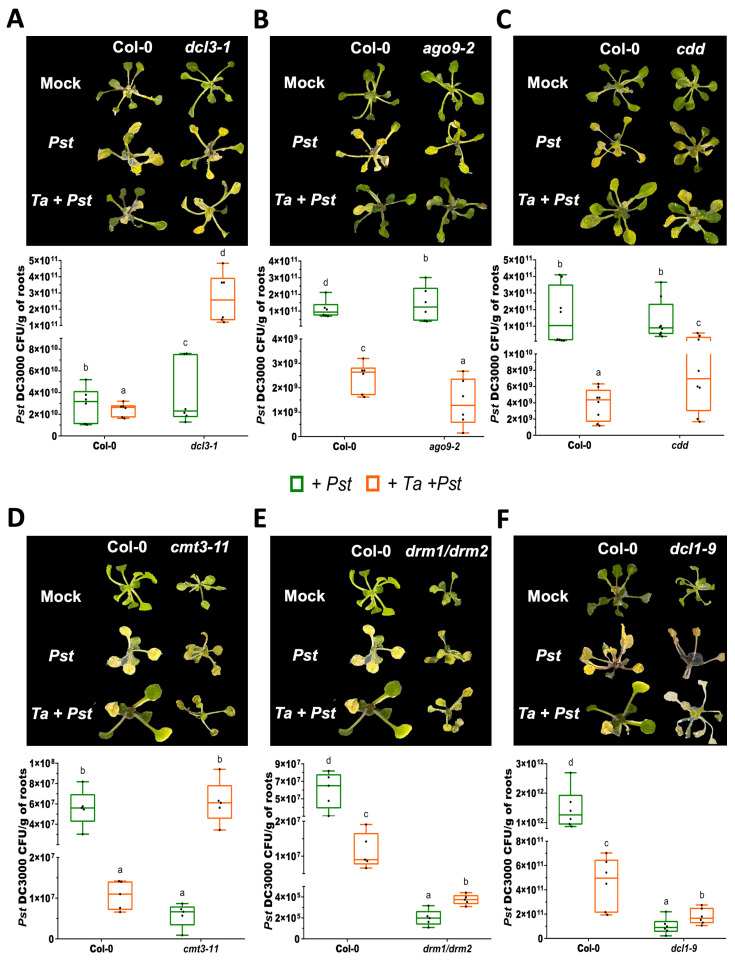
Disease symptoms and bacterial proliferation of *Pseudomonas syringae* DC3000 in Arabidopsis mutants affected in RNA silencing and DNA methylation pathways. Ten-day-old Arabidopsis plants were root-pre-treated with *T. atroviride* conidia (*Ta*) or mock-treated with buffer. At 72 hpt, aerial tissues were challenged with *Pseudomonas syringae* pv. *tomato* DC3000 (*Pst*). Representative images were taken at 5 days post-infection (dpi) for the Col-0, *dcl3-1* (**A**), *ago9-2* (**B**), *cdd* (**C**), *cmt3-11* (**D**), *drm1/drm2* (**E**) and *dcl1-9* (**F**) lines. Bacterial proliferation was quantified as colony-forming units (CFU) per gram of fresh tissue. The *cdd* triple mutant corresponds to combined mutations in *CMT3*, *DRM1*, and *DRM2*. Experiments were independently repeated twice with similar results, using 40 plants per treatment. Error bars represent standard deviation (SD). Different letters indicate statistically significant differences among the treatments as determined by one-way ANOVA followed by Tukey’s multiple comparison test (*p* < 0.05).

As expected, the *T. atroviride* pre-treatment significantly reduced disease severity and bacterial growth in the Col-0 plants (Figure 4). Notably, the *dcl3-1* and *ago9-2* mutants displayed increased lesion development and higher CFU levels under mock conditions, indicating that DCL3 and AGO9 act as positive regulators of immunity to *Pst* DC3000. While *ago9-2* showed an improved response to *T. atroviride*, with reduced damage and bacterial load upon pre-treatment compared to Col-0, *dcl3-1* exhibited exacerbated susceptibility under the same conditions, indicating that DCL3 is required for the beneficial effects of *T. atroviride* (Figure 4A,B). The *cdd* mutant displayed slightly enhanced basal resistance compared to Col-0 (Figure 4B,C). Upon the *T. atroviride* treatment, *cdd* showed a moderate ISR response; however, this response was weaker than in Col-0 and markedly less pronounced than in *ago9-2*. This suggests that although the combined loss of CMT3, DRM1, and DRM2 confers partial basal resistance, it compromises the full expression of induced systemic resistance.

Interestingly, the *cmt3-11* and *drm1/drm2* mutants exhibited stronger basal resistance than *cdd* under mock conditions (Figure 4C–E), suggesting that individual *de novo* methyltransferases may contribute to susceptibility during *Pst* DC3000 infection. However, the *T. atroviride* pre-treatment led to increased susceptibility in both lines: *cmt3-11* became more susceptible than Col-0, and *drm1/drm2*, although less severely affected, also exhibited increased disease symptoms (Figure 4D,E). These results indicate that CMT3, DRM1, and DRM2 are required for effective *T. atroviride*-induced protection against *Pst* DC3000.

The *dcl1-9* mutant displayed enhanced resistance to *Pst* DC3000 under mock conditions, consistent with a negative role for DCL1 in basal defense. However, the *T. atroviride* pre-treatment increased lesion development and bacterial growth (Figure 4F), indicating that DCL1 is required for the protective effect conferred by *T. atroviride*. Altogether, these results support a model in which DCL3 and AGO9 act as positive regulators of basal immunity, whereas DCL1 and de novo DNA methyltransferases function as negative regulators under basal conditions but are required for the establishment of *T. atroviride*-induced systemic resistance.

The increased susceptibility observed in multiple mutants following the *T. atroviride* pre-treatment and *Pst* DC3000 infection suggests a failure in immune priming, likely associated with the disrupted RdDM and sRNA-mediated regulatory pathways. These findings underscore the context-dependent and dual roles of RNA silencing and DNA methylation components in balancing plant immunity and beneficial plant–microbe interactions.

### 3.5. Distinct Roles of RNA Silencing and De Novo DNA Methyltransferases in Regulating Salicylic Acid Signaling and Immunity in Arabidopsis

**Figure 5:**
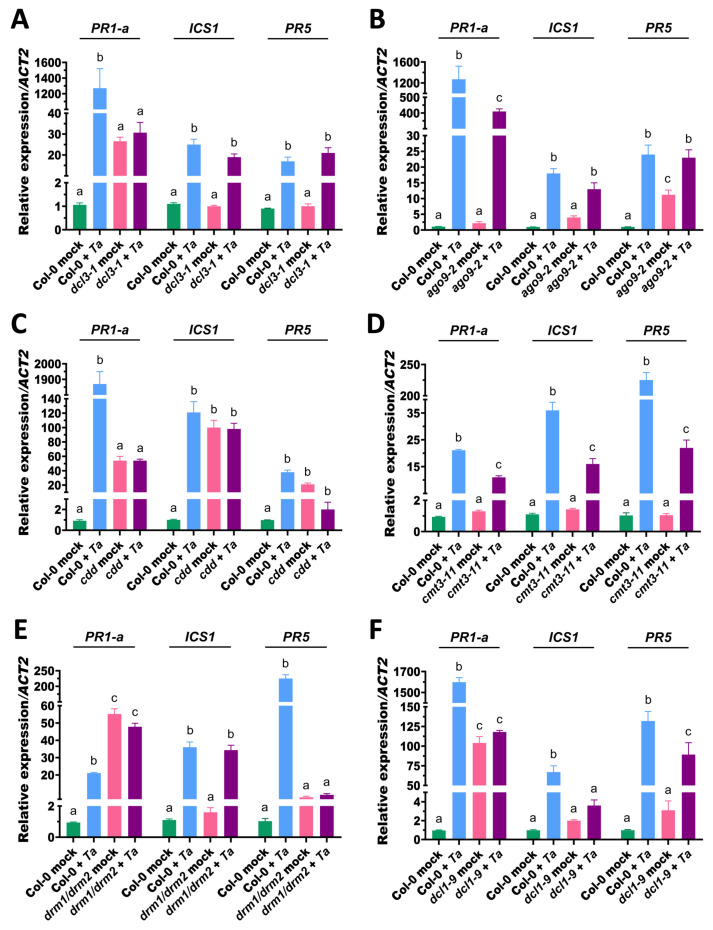
Expression of the salicylic acid (SA)-responsive marker genes *PR1-a*, *ICS1* and *PR5* in Arabidopsis mutants affected in RNA silencing and DNA methylation pathways. Ten-day-old Arabidopsis seedlings were root-treated with *T. atroviride* conidia (Ta) or mock-treated, and *PR-1a*, *ICS1* and *PR5* were quantified by RT-qPCR. Gene expression was analyzed in the *dcl3-1* (**A**), *ago9-2* (**B**), *cdd* (**C**), *cmt3-11* (**D**), *drm1/drm2* (**E**) and *dcl1-9* (**F**) lines. Expression levels were normalized to *ACT2* and calculated relative to the corresponding mock-treated control. Each genotype was analyzed in an independent experiment with its own Col-0 control, which is shown for comparison across panels and may, therefore, appear duplicated. The *cdd* mutant corresponds to the triple mutant for *CMT3*, *DRM1*, and *DRM2*. Data represent the mean ± standard deviation (SD) of two independent biological experiments, each including 40 plants per treatment. Different letters indicate statistically significant differences among the treatments as determined by two-way ANOVA followed by Tukey’s multiple range test (*p* < 0.05).

To determine whether the altered susceptibility of the *dcl3-1*, *ago9-2*, *dcl1-9*, *cdd*, *cmt3-11*, and *drm1-2/drm2-2* mutants to *Pst* DC3000 was associated with changes in salicylic acid (SA)-dependent defense signaling, we quantified the expression of *PR-1a, PR5* and *ICS1* at 72 h post *T. atroviride* treatment. In *dcl3-1*, *PR-1a* was strongly induced under mock conditions compared to Col-0, whereas *ICS1* and *PR5* remained at basal levels (Figure 5A). Upon the *T. atroviride* treatment, *PR-1a* was not further induced, in contrast to Col-0, which showed strong activation. In contrast, *ICS1* and *PR5* reached levels comparable to Col-0 (Figure 5A). This pattern suggests that it contributes to the proper coordination between upstream SA biosynthesis and downstream transcriptional responses, rather than uniformly controlling all components of the pathway.

Similarly, in *ago9-2*, only *PR5* was elevated under mock conditions. Upon *T. atroviride* treatment, all three genes were induced; however, *PR-1a* and *ICS1* remained significantly lower than in Col-0, while *PR5* reached comparable levels (Figure 5B). These results indicate that AGO9 modulates SA signaling in a gene-specific manner, allowing partial activation of downstream responses despite reduced induction of upstream components. In *cdd* mutants, *PR-1a*, *ICS1*, and *PR5* were highly expressed under mock conditions and showed no further induction after the *T. atroviride* treatment (Figure 5C). This constitutive activation suggests that the combined loss of CMT3, DRM1, and DRM2 disrupts repression of SA signaling, leading to a ceiling effect that limits further inducibility.

In *dcl1-9*, *PR-1a* was elevated under mock conditions and remained unchanged after the *T. atroviride* treatment, whereas Col-0 showed strong induction. *ICS1* was not induced in *dcl1-9*, while *PR5* was significantly upregulated following treatment (Figure 5F). This uncoupled response indicates that DCL1 constrains basal SA signaling while also being required for proper inducible activation of specific components of the pathway.

In *cmt3-11*, all three genes displayed reduced basal expression compared to Col-0 but were significantly induced upon the *T. atroviride* treatment, although to a lesser extent than Col-0 (Figure 5D). In contrast, *drm1/drm2* mutants showed elevated *PR-1a* under mock conditions, which persisted after treatment. *ICS1* expression remained comparable to Col-0, while *PR5* was not induced (Figure 5E). These results suggest that individual *de novo* methyltransferases differentially regulate basal and inducible SA-responsive genes. In *dcl1-9*, *PR-1a* was elevated under mock conditions and remained unchanged after the *T. atroviride* treatment, whereas Col-0 showed strong induction. *ICS1* was not induced in *dcl1-9,* while *PR5* was significantly upregulated following treatment (Figure 5F). This uncoupled response indicates that DCL1 constrains basal SA signaling while also being required for proper inducible activation of specific components of the pathway.

Collectively, these results demonstrate that RNA silencing and RdDM components exert non-uniform and gene-specific regulatory roles in SA signaling. DCL3 and AGO9 contribute to the coordinated activation of upstream and downstream elements, whereas DCL1 restricts basal activation while supporting inducible responses. In contrast, de novo DNA methyltransferases maintain repression of SA-responsive genes under basal conditions and enable proper transcriptional reprogramming upon microbial stimulation, thereby fine-tuning the balance between immune readiness and responsiveness.

### 3.6. Arabidopsis DCL3, AGO9, DCL1, and DNA Methyltransferases Facilitate Mutualistic Interaction with T. atroviride

To determine whether components of the RdDM pathway influence *T. atroviride* root colonization, we quantified fungal establishment in Arabidopsis mutants defective in small RNA biogenesis and DNA methylation. Based on their contrasting disease phenotypes against *B. cinerea* and *Pst* DC3000, we hypothesized that these components may also modulate beneficial plant–microbe interactions.

**Figure 6:**
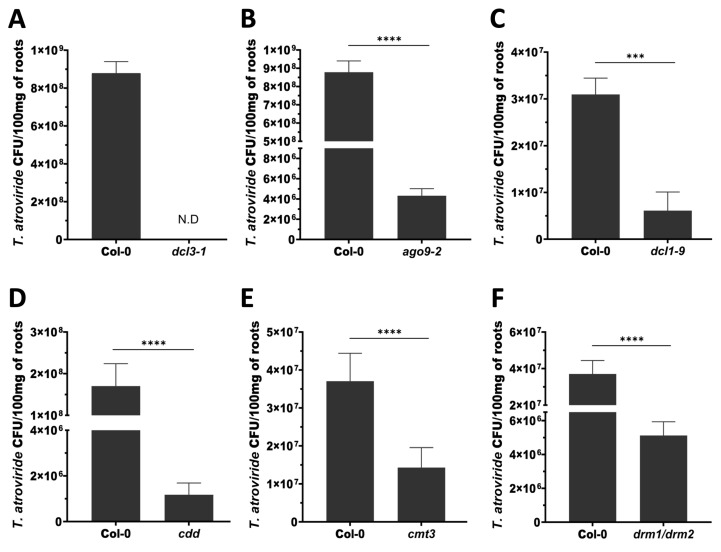
Root colonization of Arabidopsis by *Trichoderma atroviride* in mutants affected in RNA silencing and DNA methylation pathways. Ten-day-old seedlings were root-pre-treated with *T. atroviride* conidia (Ta) or mock-treated with buffer. At 48 h post-treatment (hpt), root colonization was quantified as colony-forming units (CFU) per 100 mg of fresh root tissue in the Col-0, *dcl3-1* (**A**), *ago9-2* (**B**), *dcl1-9* (**C**), *cdd* (**D**), *cmt3-11* (**E**) and *drm1/drm2* (**F**) lines. The mock-treated plants were included as controls. Data represent the mean ± standard deviation (SD) of two independent biological experiments, each including 40 plants per treatment. Asterisks indicate statistically significant differences compared to the corresponding Col-0 control, as determined by unpaired two-tailed *t*-test (*** *p* < 0.001 and **** *p* < 0.0001).

The root colonization assays conducted two days post-inoculation revealed that the *dcl3-1* mutant exhibited a drastic reduction in fungal recovery, as no CFUs were detected under the conditions tested (Figure 6A). This indicates a severe impairment in colonization capacity, although it does not exclude the possibility of low-level or transient fungal presence below the detection threshold. Similarly, the *ago9-2* and *dcl1-9* mutants showed significantly reduced CFU counts compared to Col-0 (Figure 6B,C), indicating that AGO9 and DCL1 contribute to efficient root colonization.

Consistent with previous findings, the *cdd* triple mutant exhibited impaired colonization (Figure 6D) [32]. In addition, both the *cmt3-11* single mutant and the *drm1/drm2* double mutant displayed significantly reduced CFU levels relative to Col-0 (Figure 6D,E), demonstrating that individual de novo DNA methyltransferases also contribute to fungal establishment. Together, these results indicate that multiple components of the RdDM pathway, including DCL3, AGO9, DCL1, CMT3, DRM1, and DRM2, are required for efficient colonization of Arabidopsis roots by *T. atroviride*. These findings support a model in which epigenetic regulation is not only involved in immune signaling but also in the establishment and maintenance of beneficial plant–microbe interactions.

## 4. Discussion

**Figure 7:**
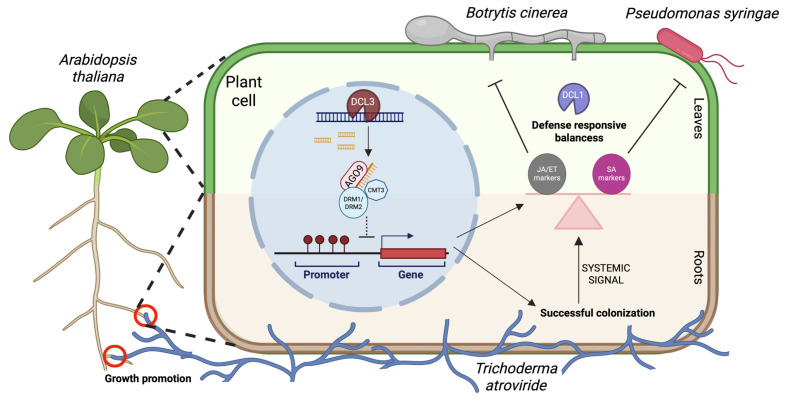
Proposed model of epigenetic regulation underlying Arabidopsis–*T. atroviride* interactions and systemic resistance. RNA-directed DNA methylation (RdDM) components modulate the balance between basal defense activation and inducible systemic responses during plant–microbe interactions. In roots, DCL3, AGO9, DCL1 and the de novo DNA methyltransferases (CMT3, DRM1, and DRM2) contribute to successful colonization by *T. atroviride*, with DCL3 playing a central role in fungal accommodation. Following root colonization, systemic signals influence defense responses in aerial tissues, where epigenetic regulators modulate the transcriptional responsiveness of the jasmonic acid/ethylene (JA/ET; e.g., *PDF1.2* and *LOX2*) and salicylic acid (SA; e.g., *PR-1a* and *ICS1*) pathways. DCL3 and AGO9 contribute to the regulation of defense-associated gene expression, while DNA methyltransferases maintain the balance between basal activation and inducibility. DCL1 is required for proper coordination of transcriptional responses underlying systemic resistance. Together, these components regulate the dynamic balance between compatibility with beneficial microbes and effective immune responses against pathogens such as *Botrytis cinerea* and *Pseudomonas syringae* pv. tomato DC3000.

In this study, we show that the components of the RNA-directed DNA methylation (RdDM) pathway and RNA silencing machinery contribute to the coordination of growth, immune responses, and beneficial plant–microbe interactions in the *Arabidopsis thaliana–Trichoderma atroviride* system (Figure 7). Rather than acting solely as positive or negative regulators of specific processes, our results indicate that these components modulate the plant’s capacity to respond dynamically to microbial cues, influencing root colonization, growth promotion, and systemic resistance. Our findings further reveal that sRNA-processing factors (DCL1, DCL3, and AGO9) and de novo DNA methyltransferases (CMT3, DRM1, and DRM2) exert distinct and context-dependent effects on plant growth and immune regulation [11,34]. Consistent with previous reports of *Trichoderma*-mediated growth promotion [15,18,19,24,35], loss of DCL3 and AGO9 resulted in increased vegetative growth under mock conditions, suggesting that these factors may act as modulators of growth under specific environmental conditions. However, these mutants did not display enhanced responsiveness to *T. atroviride*, indicating that elevated basal growth may be associated with a reduced capacity for further stimulation under these conditions. In contrast, the methyltransferase-deficient mutants (*cdd, cmt3-11,* and *drm1/drm2*) exhibited reduced growth and failed to respond to the fungal treatment. These observations are consistent with the role of de novo methylation in enabling proper growth responses to beneficial microbes [36,37], although this contribution is likely influenced by developmental stage and environmental variables. Whether this reflects altered perception, signal transduction, or downstream transcriptional regulation remains to be determined.

While targeted RT-qPCR analyses enabled the assessment of key ISR- and SAR-associated marker genes across multiple mutant backgrounds, this approach does not capture the full extent of transcriptional reprogramming underlying plant responses to both beneficial and pathogenic interactions. Transcriptome-wide approaches such as RNA sequencing (RNA-seq) would provide a more comprehensive view of the regulatory networks controlled by RdDM components, including additional responsive genes, pathway crosstalk between JA/ET- and SA-dependent signaling, and potential regulatory hubs. Future studies integrating RNA-seq with epigenomic profiling will be essential to resolve how chromatin-based mechanisms coordinate growth, immunity, and responsiveness to beneficial microbes at a systems level.

Among the sRNA-processing factors, the *dcl3-1* plants exhibited a severe reduction in root colonization, as no CFUs were recovered under the conditions tested, indicating a strong impairment in fungal establishment [11,24,37]. Under basal conditions, *dcl3-1* showed enhanced resistance to *B. cinerea* [23,24,38] but increased susceptibility to *Pseudomonas syringae* pv. *tomato* DC3000, consistent with elevated expression of JA/ET-responsive markers (*PDF1.2*, *LOX2*, and *PR4*) and increased *PR-1a* levels. However, the *T. atroviride* pre-treatment did not enhance *PR-1a* induction in *dcl3-1* and was associated with increased susceptibility to bacterial infection, suggesting an impaired ability to coordinate defense-related transcriptional responses [23,39]. These observations are consistent with an altered balance between the JA/ET- and SA-associated signaling outputs, although direct measurements of hormone levels will be required to confirm this interpretation. Together, these results support a role for DCL3 in integrating transcriptional responses associated with defense and beneficial interactions, potentially through epigenetic regulatory mechanisms that influence the plant’s responsiveness to microbial signals.

The *ago9-2* line exhibited a marked reduction in root colonization, as reflected by lower CFU recovery compared to Col-0. The induction of AGO9 during *T. atroviride* interaction [24], together with similar colonization phenotypes reported for *ago4-2*, *ago6-2*, and *ago8-1* mutants [24], suggests that members of the AGO4/6/8/9 clade may facilitate beneficial fungal establishment [40,41,42], although their specific roles remain to be fully resolved. Phenotypically, *ago9-2* shared similarities with *dcl3-1*, including enhanced basal resistance to *B. cinerea* [23,24,38] and increased susceptibility to *Pst* DC3000 under mock conditions. Upon the *T. atroviride* treatment, *PR-1a* induction in *ago9-2* was lower than Col-0, whereas *ICS1* and *PR5* reached comparable levels. This differential transcriptional response is consistent with a partial uncoupling of SA-associated signaling outputs [43]. Interestingly, despite its increased basal susceptibility, *ago9-2* displayed an improved response to the *T. atroviride* pre-treatment compared to Col-0, suggesting that AGO9 influences the balance between basal defense and inducible protection. These observations are consistent with a role for AGO9 in modulating SA-related transcriptional responses, although further analyses will be required to determine whether this regulation occurs at the level of chromatin, small RNA pathways, or both.

The *cdd* triple mutant (*cmt3/drm1/drm2*) also exhibited reduced colonization, indicating that the combined activity of CMT3, DRM1, and DRM2 contributes to efficient fungal establishment. Phenotypically, *cdd* plants displayed reduced growth and enhanced basal resistance to both pathogens, accompanied by elevated expression of JA/ET-associated (*PDF1.2*, *LOX2*, and *PR4*) and SA-associated (*PR-1a*, *ICS1*, and *PR5*) marker genes [39,44,45,46]. However, *T. atroviride* pre-treatment did not further enhance protection in *ccd* and the magnitude of induced resistance was lower than in Col-0. This pattern is consistent with a constitutively elevated defense state that limits the dynamic range of further induction. Together, these observations suggest that de novo DNA methyltransferases contribute to maintaining a balance between basal defense activation and inducibility, preventing constitutive responses from constraining the plant’s capacity to reprogram immunity upon beneficial microbial signals [44,47].

The *cmt3-11* single mutant showed reduced root colonization and impaired ISR, indicating that CMT3 contributes to efficient fungal accommodation. Basal resistance to *B. cinerea* was comparable to that of Col-0 [23,24,38]; however, ISR was not observed following the *T. atroviride* pre-treatment. This lack of inducibility was accompanied by partial deregulation of JA/ET-associated markers under mock conditions and further induction of *LOX2* and *PR4* upon fungal treatment. In response to *Pst* DC3000, *cmt3-11* exhibited enhanced basal resistance but increased susceptibility following *T. atroviride* pre-treatment. This context-dependent behavior suggests an altered coordination between basal defense activation and inducible responses. Together, these observations are consistent with a role for CMT3 in modulating the balance between the JA/ET- and SA-associated transcriptional outputs, although further analyses will be required to determine whether this reflects direct regulation of hormonal crosstalk or downstream transcriptional control.

Together, these observations are consistent with a role for DRM1/2 in regulating the balance between basal activation and inducible defense responses, potentially through epigenetic mechanisms that influence transcriptional responsiveness, although direct evidence at the chromatin level will be required to confirm this possibility.

Similarly, the *drm1/drm2* mutants displayed reduced root colonization and enhanced basal resistance to *B. cinerea* [23,24,38], but failed to develop effective ISR. In contrast to *cmt3-11*, the JA/ET-associated markers were strongly induced upon the *T. atroviride* treatment, in cases exceeding the Col-0 levels. However, this increased transcriptional response did not translate into enhanced resistance to *Pst* DC3000; instead, disease symptoms were aggravated, although to a lesser extent than in Col-0. Analysis of the SA-associated markers revealed elevated *PR-1a* expression under basal conditions, while *PR5* remained unresponsive following the *T. atroviride* treatment. This pattern suggests an altered coordination among the SA-related transcriptional outputs rather than a uniformly activated pathway. Together, these observations are consistent with a role for DRM1/2 in regulating the balance between basal activation and inducible defense responses, potentially through epigenetic mechanisms that influence transcriptional responsiveness, although direct evidence at the chromatin level will be required to confirm this possibility.

Finally, the *dcl1-9* mutants exhibited reduced root colonization, indicating that DCL1 contributes to efficient fungal establishment [24,48]. Basal resistance to *B. cinerea* was comparable to Col-0, but ISR was only partially effective. This was reflected in a differential JA/ET transcriptional response, where *PDF1.2* was strongly induced, while *PR4* reached levels similar to the Col-0 levels, suggesting an unbalanced activation of this pathway. The SA-associated responses were also altered, as *PR-1a* and *ICS1* failed to be induced following the *T. atroviride* treatment. Rather than indicating a complete impairment of SA signaling, these results point to a reduced inducibility of key regulatory components. Upon challenge with *Pseudomonas syringae* pv. tomato DC3000, the *T. atroviride* pre-treatment led to increased susceptibility compared to the untreated *dcl1-9* plants, although disease severity remained lower than in the Col-0 mock controls. This intermediate phenotype suggests that basal defense and inducible protection are partially uncoupled in the absence of DCL1. Together, these findings support a role for DCL1 in coordinating the amplitude and balance of transcriptional responses associated with systemic resistance, rather than acting as a simple positive regulator of immunity.

Collectively, these results indicate that RdDM components and the associated chromatin regulators play a dual role in modulating plant–microbe interactions, restricting excessive pathogen proliferation while enabling beneficial fungal colonization [23,39]. The phenotypic spectrum observed, ranging from complete loss of colonization in *dcl3-1* to partial defects in other mutants, highlights that sRNA-processing factors and DNA methyltransferases contribute through both distinct and partially overlapping functions.

Rather than acting as simple positive or negative regulators of immunity, these components appear to regulate the balance between basal activation and inducible defense responses. This balance is critical for maintaining compatibility with *Trichoderma*, as excessive basal activation or impaired inducibility both compromise effective systemic protection. These findings support a model in which epigenetic regulation modulates the responsiveness of defense pathways, allowing plants to accommodate beneficial microbes while retaining the capacity to mount effective immune responses.

## 5. Conclusions

This study demonstrates that RNA-directed DNA methylation (RdDM) components contribute to the coordination of plant growth, immunity, and compatibility with beneficial fungi. Our findings show that DCL3, AGO9, DCL1, and the de novo DNA methyltransferases CMT3, DRM1, and DRM2 participate in distinct yet interconnected processes that shape Arabidopsis–*Trichoderma* interactions and systemic defense responses.

DCL3 appears to be required for efficient fungal accommodation and is associated with the regulation of defense-related transcriptional responses, whereas AGO9 likely modulates immune outputs in a pathogen lifestyle-dependent manner. De novo DNA methyltransferases are necessary to maintain proper regulation of the balance between basal activation and inducible defense responses, and DCL1 contributes to the coordination of transcriptional programs underlying systemic resistance. The contrasting phenotypes observed across mutants support a functional differentiation within the RdDM machinery, where these components act as regulators of defense responsiveness rather than as simple silencers.

Collectively, these results support a model in which epigenetic pathways dynamically modulate the balance between basal defense, inducible systemic resistance, and compatibility with beneficial microbes, while also shaping growth outcomes in a context-dependent manner. This work underscores the importance of chromatin-based regulation in shaping plant responses to both pathogenic and mutualistic interactions (Figure 7).

## Data Availability

The original contributions presented in this study are included in the article/Supplementary Materials. The original data presented in the study are openly available in Repositorio IPICYT at http://hdl.handle.net/11627/6763. Further inquiries can be directed to the corresponding author.

## Supplementary Materials

The following supporting information can be downloaded at https://www.mdpi.com/article/10.3390/microorganisms14040914/s1. Table S1: Primers used for RT-qPCR and normalization with the housekeeping gene.

## Abbreviations

The following abbreviations are used in this manuscript:

ABRC	Arabidopsis Biological Resource Center
*ACT2*	ACTIN-2 gene
AGO9	ARGONAUTE 9
*cdd*	*cmt3-11/drm1-2/drm2-2*
cDNA	Complementary DNA
CFU	colony-forming units
*cmt3-11*	*CHROMOMETHYLASE 3* single mutant
Col-0	*Arabidopsis thaliana* ecotype Columbia
CTAB	cetyltrimethylammonium bromide
*dcl1-9*	*DICER-LIKE 1* mutant deficient in miRNA biogenesis
DCL3	DICER-LIKE 3
dpi	Days post-inoculation
dpt	Days post-treatment
DRM1	DNA CYTOSINE-5-METHYLTRANSFERASE 1
DRM2	DNA CYTOSINE-5-METHYLTRANSFERASE 2
ET	Ethylene
ETI	Effector-Triggered Immunity
ETS	Effector-Triggered Susceptibility
hpt	h post-treatment
HR	hypersensitive response
*ICS1*	Code for ISOCHORISMATE SYNTHASE 1 and is used as an SA marker
ISR	Induced Systemic Resistance
JA	Jasmonic Acid
*LOX2*	LYPOXYGENASE 2 gene and JA marker
MAMPs	Microbe-Associated Molecular Patterns
MS	Murashige and Skoog
nt	nucleotides
PAMPs	Pathogen-Associated Molecular Patterns
PBS	phosphate-buffered saline
PDA	Potato Dextrose Agar
*PDF1.2*	Code for PLANT DEFENSIN 1.2 gene and is used as a JA/ET marker
PGPF	plant growth-promoting fungi
Pol IV	RNA POLYMERASE IV
Pol V	RNA POLYMERASE V
*PR4*	Code for PATHOGENESIS-RELATED PROTEIN 4 and is used as an ET marker
*PR5*	Code for Thaumatin-like protein and is used as an SA response
PRRs	Pattern Recognition Receptors
PTI	PAMP-Triggered Immunity
RdDM	RNA-directed DNA methylation
RDR2	RNA-DEPENDENT RNA POLYMERASE 2
RT-qPCR	Quantitative reverse transcription PCR
SA	Salicylic Acid
SAR	Systemic Acquired Resistance
SD	Standard deviation
sRNAs	small RNAs
