# Supplementary material for: RdDM-Dependent Epigenetic Regulation Coordinates Systemic Immunity and Compatibility with Trichoderma atroviride in Arabidopsis thaliana
Source: Microorganisms. 2026 Apr 18;14(4):914. doi: 10.3390/microorganisms14040914 (PMC13119056; doi:10.3390/microorganisms14040914)
Supplement: Supplementary file 1 [file microorganisms-14-00914-s001.zip › Supplementary Table S1.pdf]

Supplementary Table S1.

**Table S1.** Primers used for RT-qPCR and normalization with the housekeeping gene.		
**Gene**	**Forward (5´- 3´) sequence**	**Reverse (5´- 3´) sequence**
**At_PR1a**	CGTCTCCGCCGTGAACAT	CGTGTTCGCAGCGTAGTTGT
**At_ICS1**	CGTGACCTTGATCCTTTCTC	ACAGCGATCTTGCCATTAG
**At_PR5**	AGCCTCGTAGATGGTTACA	GACACAGCCTGCGTATTT
**At_PDF1.2**	GGAAGACATAGTTGCATGATCC	CACCCTTATCTTCGCTGCTC
**At_PR4**	CGCCACCTACCATTTCTATAAT	CCATGCGTACGGCTTATC
**At_LOX2**	TCCTCATTTCCGCTACACCAT	CCTCCGTTGACAAGACTTTGG
**At_ACT2**	TTGCACCACCTGAAAGGAAGTA	GCTGAGGGAAGCAAGAATGG
